# The Effectiveness and Safety of Utilizing Mobile Phone–Based Programs for Rehabilitation After Lumbar Spinal Surgery: Multicenter, Prospective Randomized Controlled Trial

**DOI:** 10.2196/10201

**Published:** 2019-02-20

**Authors:** Jingyi Hou, Rui Yang, Yaping Yang, Yiyong Tang, Haiquan Deng, Zhong Chen, Yanfeng Wu, Huiyong Shen

**Affiliations:** 1 Department of Orthopedics Sun Yat-sen Memorial Hospital Sun Yat-sen University Guangzhou China; 2 Guangdong Provincial Key Laboratory of Malignant Tumor Epigenetics and Gene Regulation, Breast Tumor Center Sun Yat-sen Memorial Hospital Sun Yat-sen University Guangzhou China; 3 Department of Orthopedics Guangxi Region People's Hospital Nanning China; 4 Department of Biotherapy Center Sun Yat-sen Memorial Hospital Sun Yat-sen University Guangzhou China; 5 Department of Orthopedics 8th Affiliated Hospital of Sun Yat-sen University Sun Yat-sen University Shenzhen China

**Keywords:** mobile phone, low back pain, rehabilitation

## Abstract

**Background:**

Rehabilitation is crucial for postoperative patients with low back pain (LBP). However, the implementation of traditional clinic-based programs is limited in developing countries, such as China, because of the maldistribution of medical resources. Mobile phone–based programs may be a potential substitute for those who have no access to traditional rehabilitation.

**Objective:**

The aim of this study was to examine the efficacy of mobile phone–based rehabilitation systems in patients who underwent lumbar spinal surgery.

**Methods:**

Patients who accepted spinal surgeries were recruited and randomized into 2 groups of rehabilitation treatments: (1) a mobile phone–based eHealth (electronic health) program (EH) or (2) usual care treatment (UC). The primary outcomes were (1) function and pain status assessed by the Oswestry Disability Index (ODI) and (2) the visual analog scale (VAS). Secondary outcomes were (1) general mental health and (2) quality of life (Likert scales, EuroQol-5 Dimension health questionnaire, and 36-item Short-Form Health Survey). All the patients were assessed preoperatively and then at 3, 6, 12, and 24 months postoperatively.

**Results:**

A total of 168 of the 863 eligible patients were included and randomized in this study. Our analysis showed that the improvement of primary outcomes in the EH group was superior to the UC group at 24 months postoperatively (ODI mean 7.02, SD 3.10, *P*<.05; VAS mean 7.59, SD 3.42, *P*<.05). No significant difference of primary outcomes was found at other time points.

A subgroup analysis showed that the improvements of the primary outcomes were more significant in those who completed 6 or more training sessions each week throughout the trial (the highest compliance group) compared with the UC group at 6 months (ODI mean 17.94, SD 5.24, *P*<.05; VAS mean 19.56, SD 5.27, *P*<.05), 12 months (ODI mean 13.39, SD 5.32, *P*<.05; VAS mean 14.35, SD 5.23, *P*<.05), and 24 months (ODI mean 18.80, SD 5.22, *P*<.05; VAS mean 21.56, SD 5.28, *P*<.05).

**Conclusions:**

This research demonstrated that a mobile phone–based telerehabilitation system is effective in self-managed rehabilitation for postoperative patients with LBP. The effectiveness of eHealth was more evident in participants with higher compliance. Future research should focus on improving patients’ compliance.

**Trial Registration:**

Chinese Clinical Trial Registry ChiCTR-TRC-13003314; http://www.chictr.org.cn/showproj.aspx?proj=6245 (Archived by WebCite at http://www.webcitation.org/766RAIDNc)

## Introduction

### Background

Low back pain (LBP) is a common health problem with a point prevalence of 15% and a lifetime incidence as high as 85%. LBP is related to disability, work loss, and also accounts for high economic costs in society. For example, total annual back pain–related costs in the United States exceed US $200 billion and are still increasing [1,2].

Surgical intervention is an important treatment for LBP [3]. Due to the growing geriatric population, the number of lumbar spinal surgeries has increased rapidly. However, functional improvement and patient satisfaction after the surgery are varied; 25.0% (49/196) of the patients who underwent interbody fusion still suffer from back pain or thigh pain [4,5]. Of those who underwent discectomies, 40.0% (86/215) still remain unsatisfied with the operation and the recurrent rate can reach as high as 12.0% (24/200) [6].

Recent evidence suggests that patients with low back pain usually have inherent muscle dysfunction, which may be exacerbated by surgeries. Thus, postoperative rehabilitation becomes very critical [7,8]. Abbott et al suggested that if the patients carried out postoperative rehabilitation 3 months after surgery, their improvements in pain and function would be superior to those without any rehabilitation program [9]. A systemic review also indicated that postoperative rehabilitation would contribute to rapid functional recovery and pain alleviation of patients after lumbar spinal surgery [10]. It is also well accepted that, in order for the rehabilitation program to be fully effective, patients have to remain adherent and achieve total completion of the program [11,12]. However, there are a few efficient and effective strategies to help patients in maintaining and completing their rehabilitation therapy. On top of that, most of the recommended rehabilitation programs are clinic-based, in which patients have to visit the clinics for a total of 8 to 12 times in the span of 6 months. Personal visits to clinics are a major setback in these programs as patients in developing countries such as China have to travel a long distance to receive their treatments because of the maldistribution of medical resources [13]. Considering the cost and time, many patients eventually opt out of the rehabilitation program.

The rapid development of mobile phone–based programs provides a new option to promote health and prevent diseases [14-16]. Quinn et al used a mobile phone–based software to provide behavioral therapy for type 2 diabetes mellitus [14]. Other studies have also proved that the internet could be a useful tool to promote weight loss [17], increase physical activity [18], and improve self-management behaviors [19].

### Objectives

This trial was conducted to investigate whether a mobile phone–based program (electronic health; eHealth), designed to provide telerehabilitation for patients with LBP, would reduce pain-related disability and improve prognosis among postoperative patients who have no access to traditional clinic-based rehabilitation.

## Methods

### Trial Design

This study was a multicenter, prospective, randomized controlled trial, approved by the Ethics Committees of the Sun Yat-sen Memorial Hospital. All the 3 hospitals that participated in this study were affiliated to the Sun Yat-sen University, where the surgery could be carried out safely and skillfully. All the patients were assessed for postoperative functional ability, pain, and general mental and health status at baseline and 3, 6, 12, and 24 months.

### Inclusion and Exclusion Criteria

The researchers were required to explain the purposes, procedures, and possible risks of the trial in detail to the patients before inclusion. Written informed consents were obtained from all patients. The inclusion and exclusion criteria are shown in Textbox 1.

### Sample Size

On the basis of previous studies, we anticipated that to have a 90% chance of detecting a between-group difference of 8 points on the Oswestry Disability Index (ODI) and declaring it statistically significant using a two-sided alpha=.05, an enrollment of 168 patients was required. This calculation allowed for a loss to follow-up of 23% [11].

### Randomization

After completing the baseline survey, each participant was randomly allocated in a 1:1 ratio to the mobile phone–based eHealth program (EH) group or usual care treatment (UC) group according to a computer-generated randomization list. The allocation was stratified by a surgeon, operative procedures, and preoperative diagnosis. An email was sent to participants to inform them about their group assignment. The allocation sequence was concealed from the researchers enrolling and assessing patients.

**Inclusion and exclusion criteria for the study.**

Inclusion criteria

- Aged between 18 and 64 years
- Agreed to receive lumbar spinal surgery and that the surgical intervention involved no more than 3 columns
- Diagnosed as lumbar disc herniation, spinal stenosis, or lumbar spondylolisthesis with imaging support
- Living at least 100 km or a 2 hours’ drive away from the hospitals
- Signed the informed consent

Exclusion criteria

- Diagnosed as tuberculosis and tumor patients
- Those who accepted lumbar surgeries before this trial
- Patients with rheumatoid arthritis or ankylosing spondylitis
- Pregnancy
- Those who could not sign the informed consent or complete the rehabilitation exercise because of mental retardation or other reasons

### Intervention

#### Usual Care

No specific rehabilitation program was provided to patients randomized to the UC control group. The relevant surgeons’ usual practice was still provided, including advices to keep physically active and simple instructions to train the back muscles. Analgesia and other symptomatic treatments were also provided when necessary. All the postoperative regimes were documented.

#### eHealth

Besides the relevant surgeons’usual practice, patients randomized to the intervention group received telerehabilitation provided by eHealth, a mobile phone–based system developed by our group.

**Figure 1:**
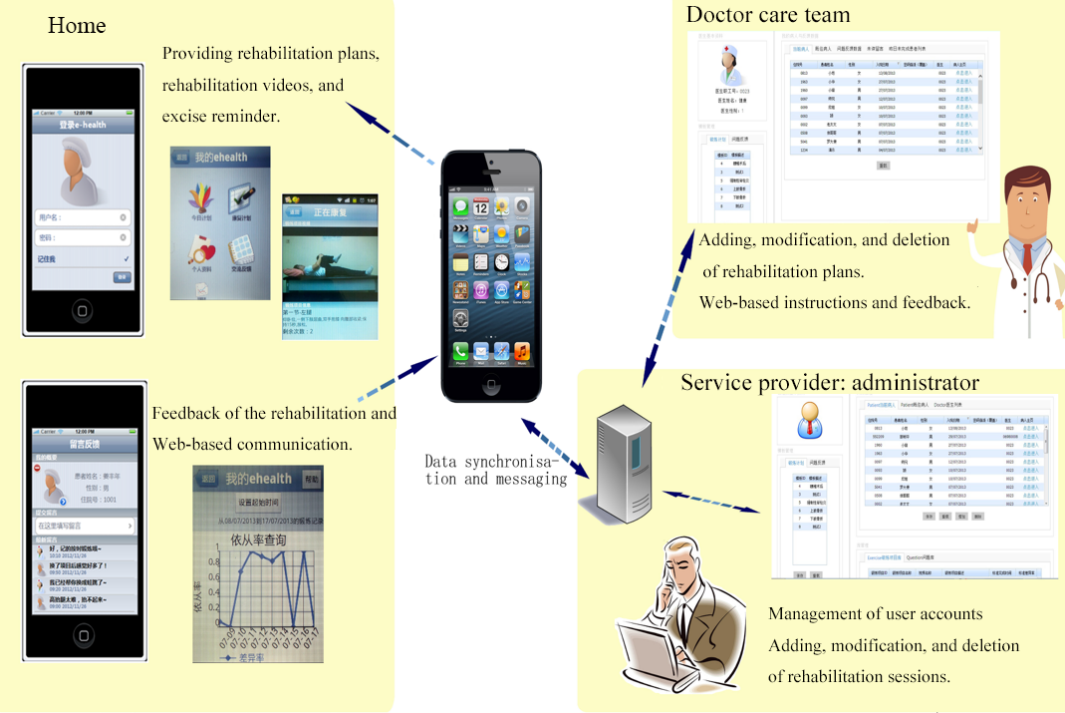
The eHealth system contained 2 interfaces: mobile phone–based interface and Web-based interface. Through mobile phone–based interface, patients were able to view the rehabilitation plans and conduct their rehabilitation following the video instructions. Daily reports and an alert were sent to prompt them to return to this system. They could also communicate with doctors through this system. Through Web-based interface, the doctors could adjust rehabilitation plans for patients and view reports about their daily exercise. All data were synchronized and stored in a remote server.

Moreover, eHealth was designed based on the user-centered theory, aimed to provide a platform for the delivery of self-management interventions [20,21]. It contained 2 interfaces: a mobile phone–based interface for patients and a Web-based interface for doctors. Through the mobile phone–based interface, patients were able to view the rehabilitation plans made by their physicians and conduct their rehabilitation following the video instructions. In addition, patients could receive daily reports about their exercise and alerts to prompt them to return to this system. They could also communicate with their doctors through this system. Through the Web-based interface, the doctors could adjust rehabilitation plans for patients and view reports about the patients’ daily exercise. All data were synchronized and stored in a remote server. The eHealth system diagram is presented in Figure 1.

The exercises included in this software were designed based on core stability exercise principles, which were all aimed to restore normal muscle strength and mobility, to activate the deep core musculature and to promote balance and coordination of the patients’ daily movements. The detailed plan of rehabilitation is shown in Multimedia Appendix 1.

The validation study was conducted with 10 healthy adults. Then, the information for the usability of the system was collected through paper-based questionnaires, 1 day after the tryout. The validation study confirmed that our system was well designed and easy to use, and the rehabilitation guidance was easy for users to understand. Combined with the results of previous studies and user preferences [11,22], our study set the rehabilitation for 20 min each time, twice a day (see Multimedia Appendix 2).

The software was installed into the patients’ phones 3 months after the surgery. Two meetings were held to show the patients how to use this software and how to conduct the exercises. The patients were also evaluated to make sure they can conduct the rehabilitation exercise correctly. They were required to complete at least 2 months of training. After 2 months, the patients could still log on to the system, and those who completed 5 or more training sessions each week were considered as high adherence, 3 to 5 training sessions as medium adherence, and 2 training sessions and less as low adherence.

### Outcome Measures

The primary outcome measures were the ODI, a disease-specific questionnaire documenting the function of known validity and reliability, and the visual analog scale (VAS) to record back pain [23,24]. The study was complemented by a series of secondary outcome measures of mental health and life status, which included the EuroQol 5-Dimension health questionnaire and 36-item Short-Form Health Survey (SF-36)—the Medical Outcomes Study SF-36 [25,26]. After baseline data collection, paper-based surveys of primary and secondary outcomes were conducted at 3, 6, 12, and 24 months. Since all outcome measurements were patient assessments, it was not possible to evaluate ODI, VAS, EQ-5D, SF-36 or Likert score blind to the randomized intervention.

At 12 months postoperative, an open survey was also conducted to detect the factors that affect patient compliance. All patients with medium and low compliance were asked to list 3 of the most important factors that they thought affected their compliance to the system.

### Statistical Methods

The analyst assessing trial outcomes was blinded to the assignments. All analyses were conducted using an intent-to-treat approach with participants analyzed according to original group assignment. The baseline data for those lost to follow-up were included. Baseline characteristics were compared between the groups using chi-square tests for categorical data and a 2-sample *t* test for continuous data. Numeric data were represented by mean (SD).

For analyses of primary and secondary outcomes, a paired *t* test was applied to examine the changes within groups. A 2-sample *t* test was applied to compare changes between the groups. Missing data were not imputed. Only available data were analyzed. Compliance rates and lost to follow-up rates were compared with groups using chi-square tests.

All the analyses were conducted using Stata version 23.0 (StataCorp LLC) and a *P*<.05 was declared as significant.

## Results

### Study Population and Follow-Up

Recruitment occurred between August 2013 and November 2014 at 3 hospitals, and 845 patients were assessed for eligibility. Of those, 428 patients were excluded for not meeting the inclusion criteria or meeting the exclusion criteria. Of the 417 eligible patients, 92 were not approached, 135 declined to participate, and 22 consented patients withdrew before randomization. The final 168 consenting patients were then randomized in this study.

**Figure 2:**
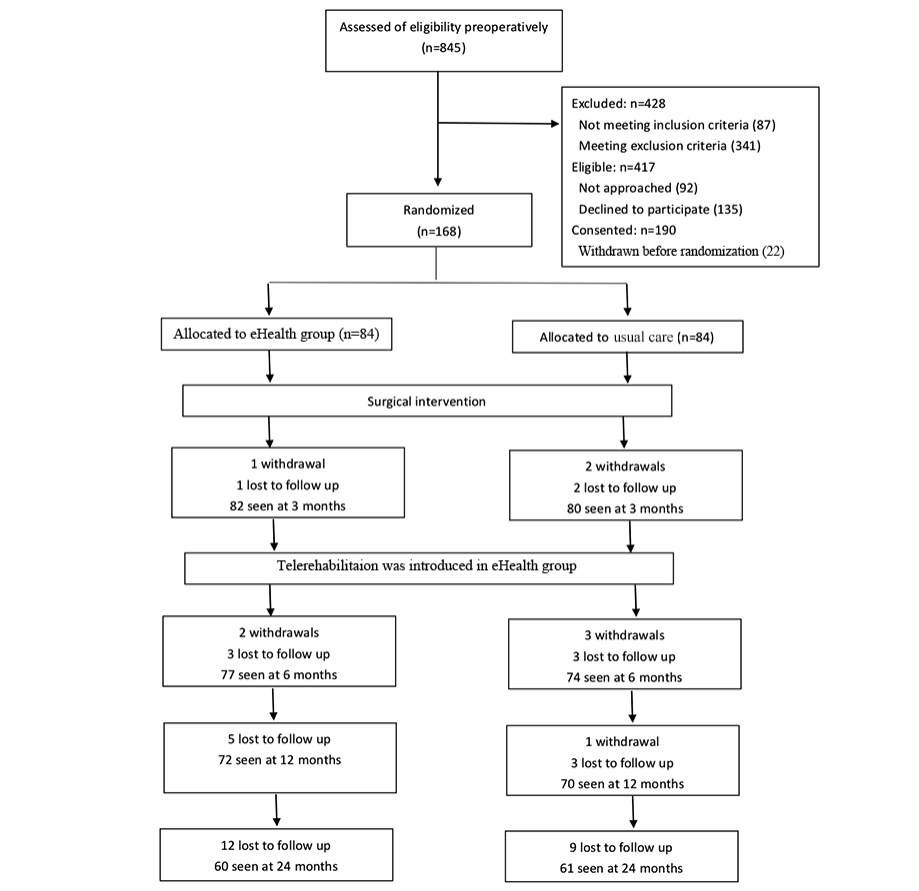
Flowchart.

All the randomized patients received operation treatments and completed the required baseline assessments. However, during the study, 2 patients in the EH group and 4 patients in the UC group dropped out at 3 months. From the EH group, 82 patients entered the treatment phase, of which 77 finished the treatment and follow-up was done at 6 months. In the UC group, 80 and 74 patients were met for follow-up at 3 months and 6 months, respectively. The follow-up rate in the EH group was 97.62% (82/84) at 3 months, 91.67% (77/84) at 6 months, 85.71% (72/84) at 12 months, and 71.43% (60/84) at 24 months. In UC group, the follow-up rate was 95.24% (80/84) at 3 months, 88.10% (74/84) at 6 months, 83.33% (70/84) at 12 months, and 72.62% (61/84) at 24 months (see Figure 2).

### Baseline Characteristics

Both the clinical and demographic characteristics of the patients were similar in the 2 groups (*P*<.05, see Table 1). Most of the study participants were married and had only finished high school or lower. On an average, participants reported moderate to severe pain and functional impairment based on ODI and VAS scores.

### Adherence to Interventions

Median eHealth attendance was 5 times per week (interquartile range, IQR, 4-6)，5 times per week (IQR 3-6), and 5 times per week (IQR 4-6) for 6, 12, and 24 months postoperatively. A total of 50, 37, and 38 patients were considered as high compliance at 6, 12, and 24 months, respectively, postoperatively. Although the high compliance rate was higher at 24 months (63.33%) compared with that at 12 months postoperatively (51.39%), it was not statistically significant (*P*>.05, see Table 2). Of these participants, 24 completed the whole trial with 6 or more training sessions each week and, therefore, were considered as the highest compliance (HC) group.

To determine the reason for low compliance, we carried out a brief survey focusing on the medium and low compliance group, asking them to list out the top 3 factors that affected their adherence at 12 months. A total of 33 out of 35 patients considered lack of communication with their doctors as the important factor. Through our record, we found that mean communication frequency was 2.54 (SD 0.89) for patients with medium or low compliance and 4.46 (SD 1.35) for patients with high compliance. The frequency of responses from doctors was significantly higher in patients with high compliance (*P*<.05). The other common reasons listed by patients were the concern about the accuracy of the action, limited symptom improvement, and lack of motivation (see Multimedia Appendix 3).

### Primary Outcomes

The ODI and VAS for the EH and UC group were similar at baseline and 3 months postoperatively. At 6 and 12 months, the mean for change of the ODI from baseline was –7.27 (SD 5.31) and –18.43 (SD 23.92),respectively, for the EH,while it was –7.90 (SD 4.53) and –14.39 (SD 4.64), respectively, for the UC group (see Table 2). No significant difference was found between the EH and UC at 6 months and 12 months (*P*>.05).

**Table 1:** Demographics and baseline characteristics of all participants.

Characteristics		UC^a^ (n=84)	EH^b^ (n=84)	*P* value
Female, n (%)		42 (50)	48 (57)	.35
Age (years), mean (SD)		49.36 (9.52)	51.11 (9.54)	.24
**Education status, n (%)**				
	High school or lower	63 (75)	60 (71)	.60
	College degree or higher	21 (25)	24 (29)	.60
	Currently employed	62 (74)	58 (69)	.50
**Marriage status, n (%)**				
	Married	76 (90)	73 (87)	.72
	Divorced	5 (6)	6 (7)	.72
	Single	3 (4)	5 (6)	.72
**Intervertebral discs involved in surger**y**, n (%)**				
	1 disc	37 (44)	36 (43)	.96
	2 discs	41 (49)	41 (49)	.96
	3 discs	6 (7)	7 (8)	.96
Mean ODI^c^ score (SD)^d^		55.40 (14.78)	54.14 (15.18)	.59
Mean VAS^e^ score (SD)^f^		60.11 (15.99)	57.71 (14.91)	.32
Mean Likert score (SD)^g^		59.14 (14.86)	59.71 (16.49)	.40
Mean EQ-5D^h^ score (SD)^i^		35.75 (15.37)	34.26 (14.84)	.32
Mean SF-36 GH^j^ score (SD)^k^		13.55 (5.58)	13.60 (6.02)	.96
Mean SF-36 PF^l^ score (SD)^m^		21.11 (8.36)	21.52 (8.72)	.75

^a^UC: usual care.

^b^EH: eHealth program.

^c^ODI: Oswestry Disability Index.

^d^Rated to assess the patient’s level of disability because of low back pain. Ranged from 0 to 100, with higher scores indicating more disability.

^e^VAS: visual analog scale.

^f^Rated between 0 and 100 with 100 representing worst pain possible.

^g^Measured using an 11-point numerical rating scale for average difficulty for movement in the previous week, where 0 indicated no difficulty and 10 indicated most difficulty.

^h^EQ-5D: EuroQol 5-Dimension health questionnaire.

^i^Rated between 0 and 100 with 100 representing a perfect health-related quality of life.

^j^SF-36 GH: General health for 36-item Short-Form Health Survey.

^k^Ranging from 0 to 100, with higher scores indicating better health-related quality of life.

^l^SF-36 PF: Physical functioning for 36-item Short-Form Health Survey.

^m^Ranging from 0 to 100, with higher scores indicating better health-related quality of life.

**Table 2:** Compliance status in different follow-ups.

Follow-up (month)	Low compliance, n (%)	Medium compliance, n (%)	High compliance, n (%)	*P* value for chi-square test
6	11 (14.47)	16 (19.74)	50 (65.79)	.23
12	8 (11.11)	27 (37.50)	37 (51.39)	.23
24	6 (11.48)	16 (26.23)	38 (62.29)	.23

**Table 3:** Primary outcomes change from baseline and between-group difference.

Measurements and follow-up (month)		UC^a^ change from baseline		EH^b^ change from baseline		Difference UC versus EH, mean (SD)	*P* value
		Participants	Mean (SD)	Participants	Mean (SD)		
**ODI^c^**							
	3	80	–7.90 (4.53)	82	–7.27 (5.31)	–0.63 (0.78)	.42
	6	74	–14.39 (4.64)	77	–18.43 (23.92)	4.0 (2.83)	.16
	12	70	–22.07 (5.56)	72	–21.58 (24.64)	–0.49 (2.98)	.87
	24	61	–23.41 (6.65)	60	–30.43 (23.75)	7.02 (3.18)	.03
**VAS^d^**							
	3	80	–7.61 (5.15)	82	–7.02 (4.45)	–0.59 (0.76)	.44
	6	74	–14.19 (5.11)	77	–17.49 (25.48)	3.30 (2.96)	.27
	12	70	–21.94 (5.8)	72	–20.55 (25.92)	–1.39 (3.13)	.66
	24	61	–22.36 (6.90)	60	–29.95 (25.60)	7.59 (3.42)	.03

^a^UC: usual care.

^b^EH: eHealth program.

^c^ODI: Oswestry Disability Index.

^d^VAS: visual analog scale.

However, at 24 months, improvement in the ODI was more significant in the EH group compared with the UC group (*P*<.05). For the VAS, the change was also not significantly different between the EH and UC at 6 months and 12 months (*P*>.05). At 24 months, the mean for change of the VAS from baseline was –22.36 (SD 6.90) in the EH and –29.95 (SD 25.60) in the UC. The improvement of the VAS was more significant in the EH than the UC (*P*<.05, see Table 3).

### Secondary Outcomes

No difference in the Likert scale for movement was found at 3, 6, and 12 months postoperatively in the EH and UC. At 24 months, patients in the EH displayed superior results of Likert scale (mean of change: EH –32.51, SD 25.94; UC –22.54, SD 5.81; *P*<.05, see Table 4).

As for the EuroQol-5 Dimension (EQ-5D), the change was similar for EH and UC at 3 months. At 6 months, the improvement for EQ-5D was 0.23 (SD 0.03) for the EH and 0.13 (SD 0.08) for the UC. The patients in the EH got a significantly superior result over that in the UC. This advantage was sustained at subsequent time points (*P*<.05, see Table 4).

For the SF-36, the improvement was more significant in the EH compared with the UC at 3, 6, and 24 months (*P*<.05, see Table 4).

### Subgroup Analysis

In the EH group, 24 patients completed all the follow-ups, with average eHealth attendance no less than 6 times per week, considered as the highest compliance. Thus, we conducted a subgroup analysis between the HC group with UC group.

Both the clinical and demographic characteristics were consistent between the 2 groups at baseline (see Multimedia Appendix 3). There were no significant differences in the changes of the ODI and VAS at 3 months. However, the HC group was superior to the UC group in the posttreatment ODI and VAS at 6 months (*P*<.05). This advantage was sustained at 12 and 24 months (see Table 5).

### Adverse Events

Adverse events, mostly mild, self-limited joint and back pain, were reported in 9 EH and 6 UC participants. It did not differ significantly in frequency or severity of adverse events in these 2 groups.

**Table 4:** Secondary outcomes change from baseline and between-group difference outcomes.

Measurements and follow-up (month)		UC^a^ change from baseline		EH^b^ change from baseline		Difference UC versus EH, mean (SD)		*P* value	
		Participants	Mean (SD)	Participants	Mean (SD)				
**Likert score**									
	3	80	–7.79 (4.96)	82	–7.20 (4.74)		0.20 (0.78)		.80
	6	74	–13.51 (5.39)	77	–19.66 (26.47)		6.15 (3.08)		.05
	12	70	–21.09 (5.68)	72	–23.00 (27.12)		1.91 (3.31)		.56
	24	61	–22.54 (5.81)	60	–32.51 (25.94)		9.98 (3.43)		.01
**EQ-5D^c^**									
	3	80	0.09 (0.02)	82	0.09 (0.02)		0.00 (0.00)		.62
	6	74	0.13 (0.08)	77	0.23 (0.03)		–0.10 (0.01)		<.001
	12	70	0.17 (0.03)	72	0.24 (0.04)		–0.05 (0.01)		.003
	24	61	0.22 (0.04)	60	0.35 (0.03)		–0.12 (0.01)		.001
**SF-36 GH^d^**									
	3	80	38.16 (2.43)	82	40.01 (3.37)		–1.85 (0.46)		.004
	6	74	45.85 (3.43)	77	54.75 (4.59)		–8.90 (0.66)		.002
	12	70	55.53 (3.86)	72	56.25 (5.31)		–0.72 (0.78)		.36
	24	61	57.98 (5.26)	60	62.80 (6.61)		–4.82 (1.09)		.002
**SF-36 PF^e^**									
	3	80	30.58 (2.29)	82	40.76 (3.05)		–2.18 (0.42)		.004
	6	74	46.35 (3.62)	77	56.12 (4.48)		–9.77 (0.66)		.003
	12	70	56.13 (4.79)	72	56.74 (5.83)		–0.61 (0.89)		.49
	24	61	59.07 (5.89)	60	62.45 (5.78)		–3.38 (1.06)		.02

^a^UC: usual care.

^b^EH: eHealth program.

^c^EQ-5D: EuroQol 5-Dimension health questionnaire

^d^SF-36 GH: General health for 36-item Short-Form Health Survey

^e^SF-36 PF: Physical functioning for 36-item Short-Form Health Survey

**Table 5:** Subgroup analysis of primary outcomes change from baseline and between-group difference outcomes.

Measurements and follow-up (month)		UC^a^ change from baseline		HC^b^ change from baseline		Difference UC versus HC, mean (SD)	*P* value
		Participants	Mean (SD)	Participants	Mean (SD)		
**ODI^c^**							
	3	80	–7.90 (4.53)	24	–7.71 (5.29)	–0.19 (1.10)	.86
	6	74	–14.39 (4.64)	24	–32.33 (25.56)	17.94 (5.24)	<.001
	12	70	–22.07 (5.56)	24	–35.46 (25.88)	13.39 (5.32)	.02
	24	61	–23.41 (6.65)	24	–42.21 (25.26)	18.80 (5.22)	.01
**VAS^d^**							
	3	80	–7.61 (5.15)	24	–6.42 (4. 91)	–1.20 (1.19)	.32
	6	74	–14.19 (5.11)	24	–33.75 (25.67)	19.56 (5.27)	.01
	12	70	–21.94 (5.8)	24	–36.29 (25.38)	14.35 (5.23)	.01
	24	61	–22.36 (6.90)	24	–43.92 (25.50)	21.56 (5.28)	.001

^a^UC: usual care.

^b^HC: highest compliance.

^c^ODI: Oswestry Disability Index.

^d^VAS: visual analog scale.

## Discussion

### Principal Findings

Much research had been done to explore the effect of rehabilitation on postoperative patients [9,27-29]. Compared with previous experiments, we aimed to explore the application of mobile phone–based rehabilitation in postoperative patients with LBP through a well-designed clinical trial. All the patients in our study lived far away from the hospital and were unable to accept the traditional clinic-based rehabilitation. This design is critical for developing countries. Due to the extremely uneven distribution of health resources, traditional clinic-based postoperative rehabilitation cannot be implemented in patients living faraway, whereas eHealth could be a solution to bridge the gap [30].

In this randomized controlled trial, we compared postoperative patients (EH group) with low back pain treated by eHealth, a mobile phone–based telerehabilitation system, with those that received nonspecific rehabilitation (UC group). We found that primary outcomes (ODI and VAS) in the EH group were superior to the UC group at 24 months postoperatively. However, no significant difference was found at all the other time points during follow-up. Furthermore, we compared 24 patients having an average eHealth attendance of no less than 6 times per week (HC group) with the UC group. Subgroup analysis showed that the improvements of the primary outcomes were more significant in the HC group compared with the UC group at 6, 12, and 24 months. These results suggest that patients with a higher compliance with our telerehabilitation system tend to have a better prognosis.

Adherence to postoperative rehabilitation in clinical practice is a serious problem [31]. Previous studies focused on clinic-based rehabilitation reported very low levels of compliance, with almost 30% failing to attend any classes, and of those attending, only 60% attended more than half of the classes [32,33]. Moreover, nonadherence of home-based rehabilitation could be as high as 50% [34]. Compared with previous studies, this study adopted video-based coaching and had a higher compliance: high compliance rates were 65.79%, 51.39%, and 62.29% at 6, 12, and 24 months, respectively. This paper supports the notion that well-designed connected health technologies, including digital, mobile health, and telehealth, could better support patients in their rehabilitation and provide an opportunity to increase adherence to exercise and rehabilitation [35].

This study also found that the 24 patients who completed 6 or more training sessions each week throughout the trial (HC group) had a better prognosis. This may be because the rehabilitation exercise needs to reach a certain length of time to achieve a more significant effect [36]. The rehabilitation protocols in previous studies were 40 to 60 min each day [11,22]. This study had set the rehabilitation for 20 min each time, twice a day, given the results of our previous validation study of user preferences (shown in Multimedia Appendix 2). However, in practice, many patients did not strictly conduct the exercise twice a day; therefore, the duration of the exercise for each day did not meet the requirements for a more efficient training session. Future research should pay more attention to the training duration of 1 single section and 1 day. Especially in the study of patients’ self-rehabilitation, the training duration should be set for longer than what we expected. Meanwhile, the compliance to rehabilitation should be improved as much as possible. However, it must be noted that noncompliance is a complicated issue and the reasons for noncompliance are multifactorial. Our survey of patients with medium and low compliance at 12 months tried to find the main reasons for noncompliance, although most patients cited insufficient communication with doctors as the main cause for noncompliance. Further analysis implied that the patients’ need for communication with doctors was because of the patients’ doubts during rehabilitation, such as whether the actions were standard, the intended goal of rehabilitation, or if more motivation was needed. All these reasons listed by the patients actually reflect that the doubt-free experience is very important for maintaining a high compliance to rehabilitation, especially for home-based rehabilitation. A well-designed system should possess the following features to create a doubt-free experience: (1) a clear goal, (2) comprehensive and detailed instructions, and (3) timely communication and motivation.

The abovementioned features could be achieved by optimizing the designs of a mobile phone–based system. Previous studies have revealed that the mobile phone is an effective tool in improving health behaviors of patients [37-39]. In a study conducted by Liu et al, patients with chronic obstructive pulmonary disease were encouraged to perform daily endurance walking while following the tempo of the music from a program installed on a mobile phone [40]. Lambert et al found that people with musculoskeletal conditions had better adherence to their home exercise programs provided on an app with remote support compared with paper handouts [41]. Meanwhile, the mobile phone can provide a lot of self-detecting methods, such as the Global Positioning System’s positioning and gravity-sensing technology to track elderly patients and provide timely feedback to the medical staff [42]. The electrocardiogram, the blood glucose meter, and the blood pressure meter connected through Bluetooth could automatically upload health-related data to the medical system [43-45]. With the advancement of technology, motion-capture devices may also be applied in telerehabilitation [46]. Until then, the compliance could be better improved with remote monitoring of the patients’ rehabilitation.

### Limitations

The main limitation of this study was the high loss to follow-up rate. Compared with previous studies, the loss to follow-up rates were similar to the loss to follow-up rates in this study [47-49]. Further analysis showed that the baseline characteristics were similar among patients who were met or lost to follow-up at 24 months (*P*>.05, see Multimedia Appendix 3). Also, no significant difference was found in the improvement of primary outcomes at 12 months between patients who were met and were lost to follow-up between 12 and 24 months both in the UC and EH group (*P*>.05, see Multimedia Appendix 3). It implied that the reason for the loss to follow-up was the patients’ own reason, which was randomized, instead of poor prognosis. The final result might not be seriously affected by those who were lost to follow-up.

The reason for the high loss to follow-up rate was probably because of the long distance from the patients’ home to the hospital, which was 1 of the inclusion criteria. These inclusion criteria are consistent with our aim, which focused on remote self-rehabilitation. However, it also adds to the difficulty for following up. As our study was conducted with paper-based questionnaires, patients included in our study had to travel a long distance back to hospitals or mail back the questionnaires, causing more to be lost to follow-up. In order to overcome this problem, electronic surveys have great potential to improve data collection [50]. A major advantage of applying electronic surveys is that they could increase the amount of data collected at a lower cost [51]. Many researchers have explored the application of electronic versions of questionnaires. Terri et al found that electronic versions of the Faces Pain Scale-Revised and the Color Analog Scale on a mobile phone demonstrated good agreement with the original paper and plastic versions of these scales [52]. Pawar et al found that the software version of the Roland-Morris Disability Questionnaire was comparable with the paper version in patients with LBP [53]. Other researchers also found that Web- or mobile device–based systems could facilitate consecutive patient data collection in randomized controlled trials and could be used to increase response rates and enhance quality of research [54,55]. Therefore, future studies should consider applying electronic tools to simplify the follow-up process and reduce loss to follow-up.

### Conclusions

In conclusion, eHealth, a mobile phone–based telerehabilitation system, may be an effective rehabilitation tool for postoperative patients with LBP, especially for those who have no access to traditional clinic-based rehabilitation. The effectiveness of eHealth was more evident in patients with higher adherence. However, more studies are still needed to find optimal methods to improve compliance.

## Appendix

Multimedia Appendix 1 The detailed plan of rehabilitation.

Multimedia Appendix 2 Results of the validation study.

Multimedia Appendix 3 Supplementary tables.

Multimedia Appendix 4 CONSORT‐EHEALTH checklist (V 1.6.1).

## Abbreviations

EH: eHealth program
eHealth: electronic health
EQ-5D: EuroQol 5-Dimension health questionnaire
HC: highest compliance
IQR: interquartile range
LBP: low back pain
ODI: Oswestry Disability Index
SF-36: 36-item Short-Form Health Survey
SF-36 GH: General health for 36-item Short-Form Health Survey
SF-36 PF: Physical functioning for 36-item Short-Form Health Survey
UC: usual care treatment
VAS: visual analog scale
