# Supplementary material for: The Effectiveness and Safety of Utilizing Mobile Phone–Based Programs for Rehabilitation After Lumbar Spinal Surgery: Multicenter, Prospective Randomized Controlled Trial
Source: JMIR Mhealth Uhealth. 2019 Feb 20;7(2):e10201. doi: 10.2196/10201 (PMC6404639; doi:10.2196/10201)
Supplement: Multimedia Appendix 1 [file mhealth_v7i2e10201_app1.pdf]

Supplementary Figure 1: Rehabilitation Protocols

Rehabilitation exercises can be divided into two categories: stretching training and strength training. Each training consisted of 4~5 actions from these two categories. Patients were asked to train 20 minutes one time, 2 times a day.

I Stretching Exercises

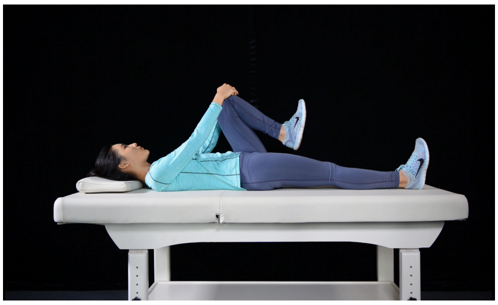

Section 1

Lie down, use your hands to flex one side of your hips, maintain 15 seconds when get to the maximum flexion, then relax.

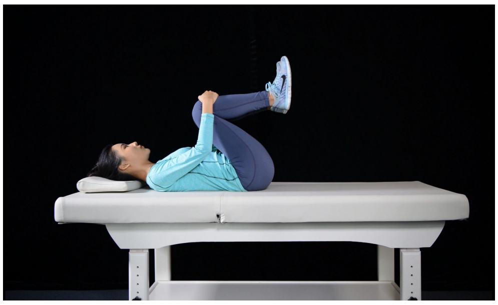

Section 2

Lie down, use your hands to flex both sides of your hips, maintain 15 seconds when get to the maximum flexion, then relax.

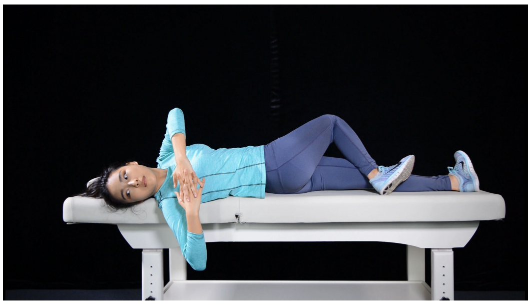

Section 3

Lie down, place your forearms in front of the chest with hands holding in each other; Then turn your upper and lower body in opposite direction as hard as possible; maintain for 15 seconds and then relax.

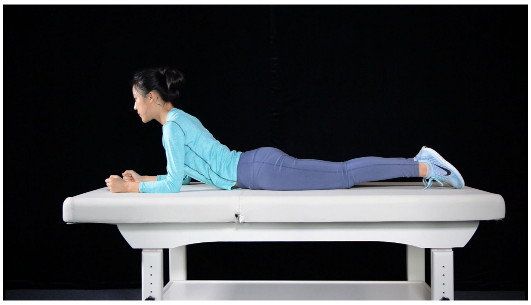

Section 4

Take prone position, raise your head and upper body with two arms flexed. stretch your waist as much as possible, maintain for 15 seconds and then relax.

II Strengh Exercises

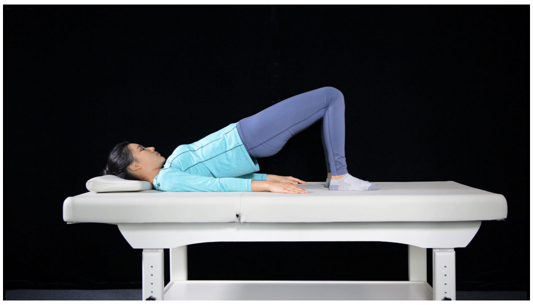

Section 5

Lie down, contract your back to lift the waist and buttock off the bed, and maintain 5S at the top. then relax.

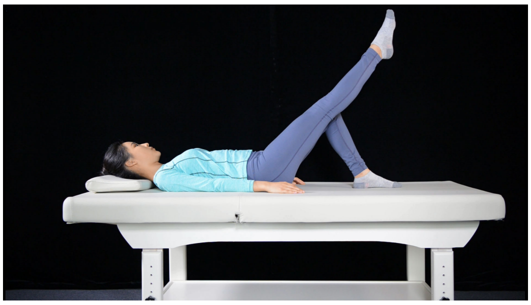

Section 6

Lie down, raise one side of your legs while another side flexed. maintain 5S at the top. then relax.

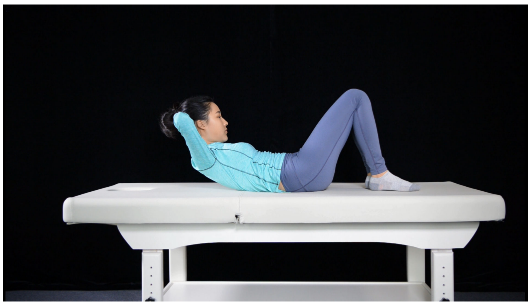

Section 7

Lie down, bend your hips and knees, hold your head with both hands, contract your abdominal muscles to lift shoulders off the bed, maintain 5S at the top. then relax.

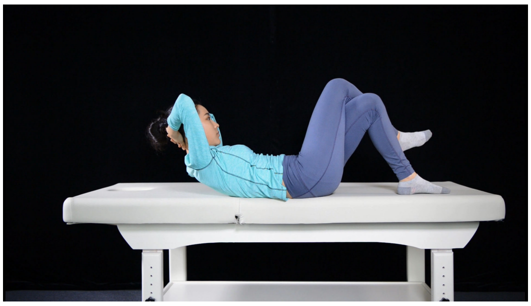

Section 8

Lie down, cross your legs, hold your head with both hands, contract your abdominal muscles to lift shoulders off the bed, maintain 5S at the top. then relax.

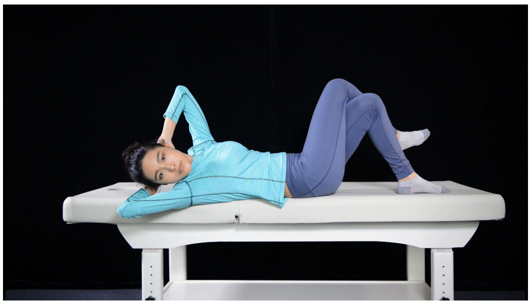

Section 9

At the top of the action in section 8, turn your upper body to one side. maintain 5S at the top. then relax.

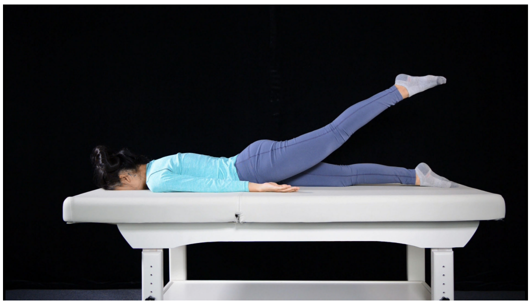

Section 10

In prone position, slowly lift one lower limb off the bed. maintain 5S at the top. then relax.

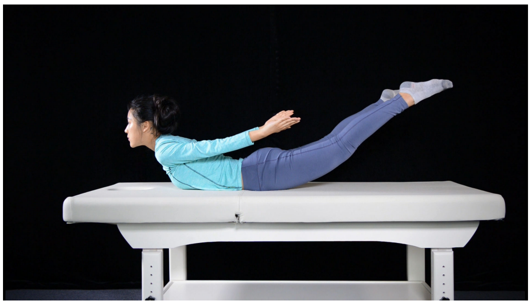

Section 11

In prone position, contract your back to lift upper body and lower limbs off the bed, maintain 5S at the top. then relax.

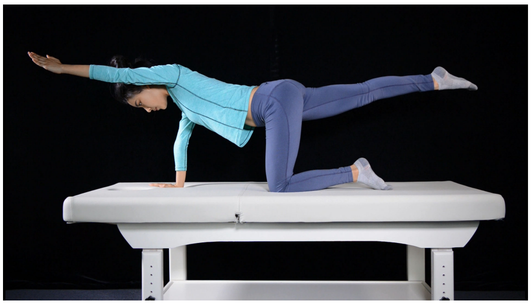

Section 12

With your hands and knees supporting, lift one side of your upper limbs and the opposite lower limb off bed. maintain 5S at the top. then relax.

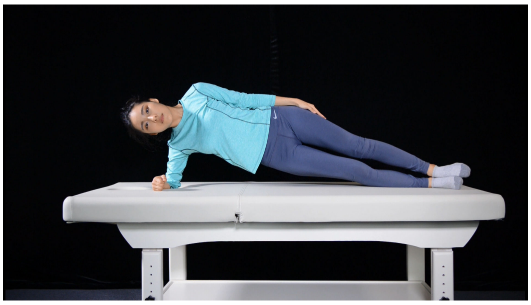

Section 13

In lateral position, with elbow and lower limb supporting, lifts the waist off the bed, and maintain 5S at the top. then relax.

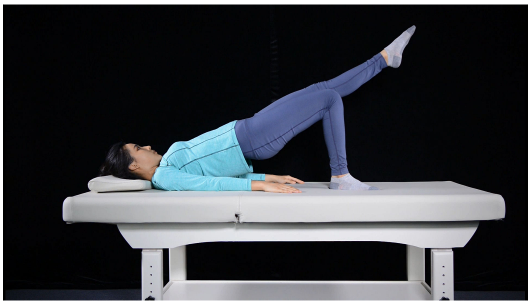

Section 14

At the top of the action in section 5, lifts one side of the lower limbs off the bed, and maintain 5S at the top. then relax.

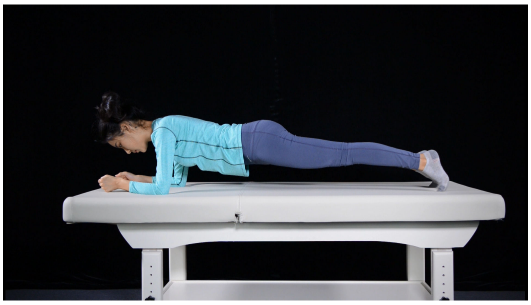

Section 15

In prone position, with elbows and feet supporting, lift the body away off the bed. maintain 5S at the top. then relax.
