# Supplementary material for: The Effectiveness and Safety of Utilizing Mobile Phone–Based Programs for Rehabilitation After Lumbar Spinal Surgery: Multicenter, Prospective Randomized Controlled Trial
Source: JMIR Mhealth Uhealth. 2019 Feb 20;7(2):e10201. doi: 10.2196/10201 (PMC6404639; doi:10.2196/10201)
Supplement: Multimedia Appendix 2 [file mhealth_v7i2e10201_app2.pdf]

Supplementary Figure 2: Questionnaire for the Validation of E-health system

1. Your gender

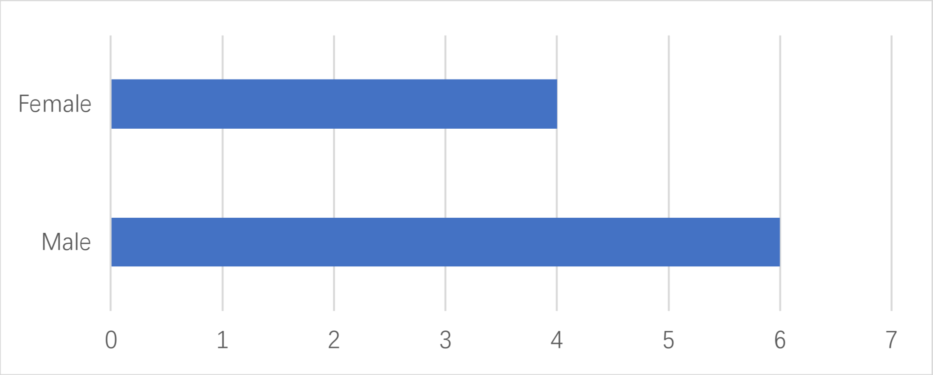

2. Your age（Mean ±SD） : 42.23±5.33

3. Your education level:

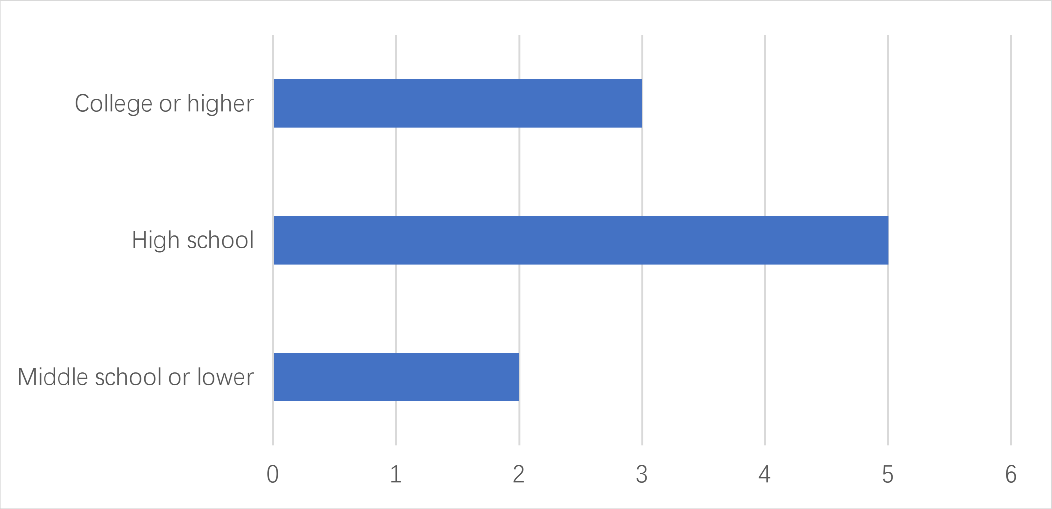

4. Do you think the design of software is reasonable?

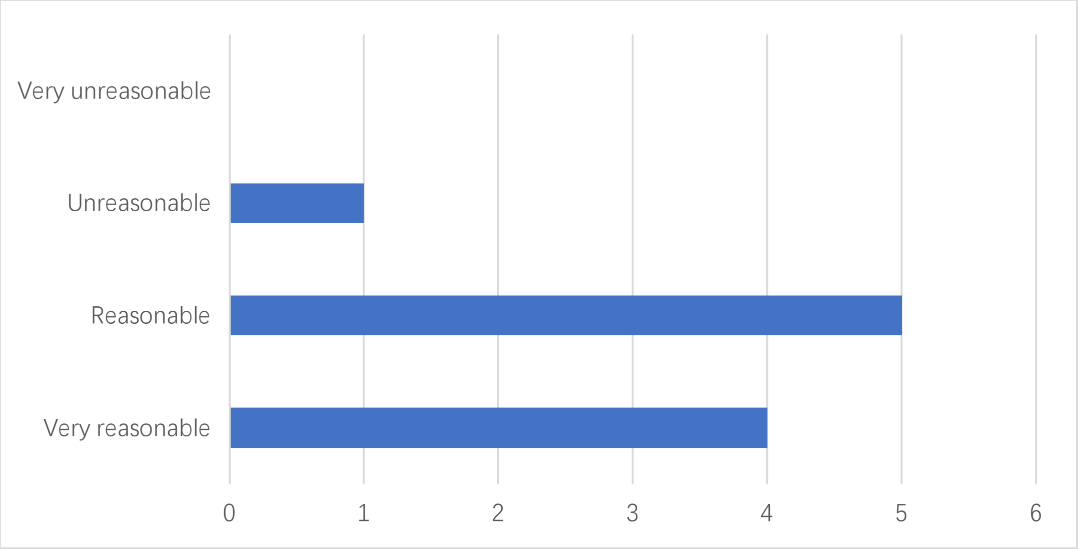

5.Do you think the content of rehabilitation guidance is easy to understand?

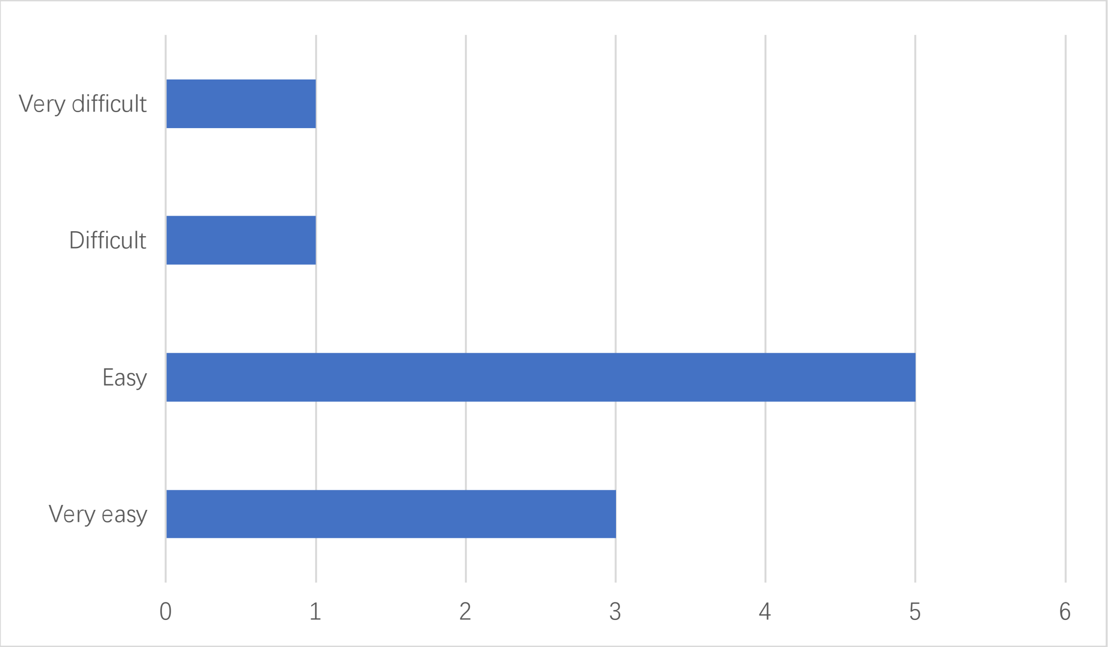

6. How difficult do you think it is to perform rehabilitation according to this system?

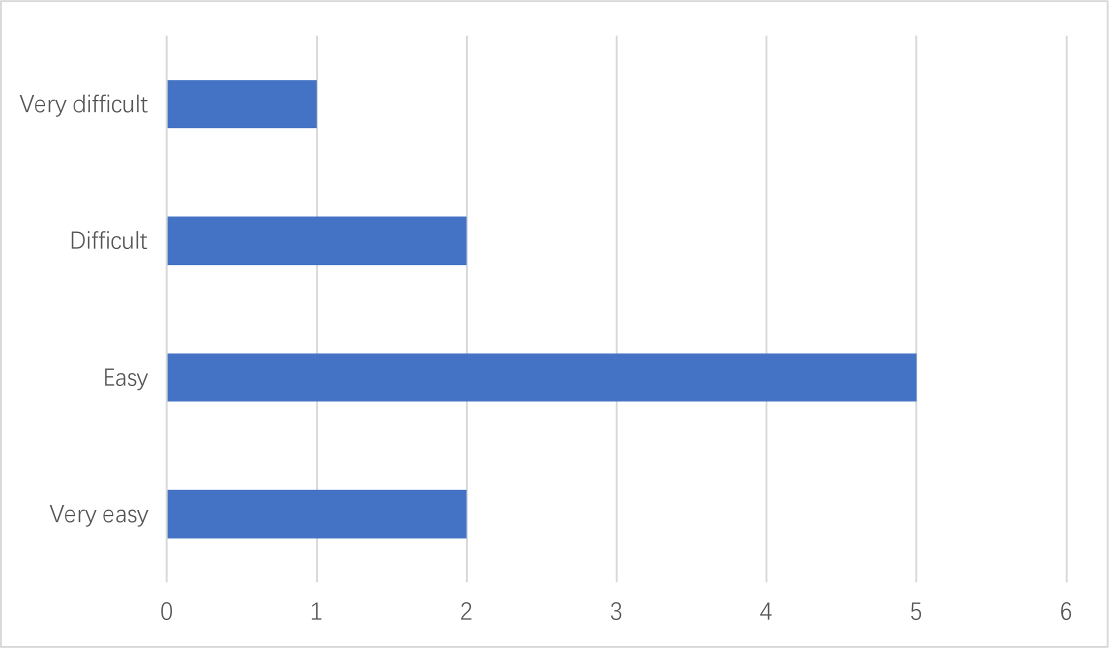

7. How much do you think our software helps to improve your compliance?

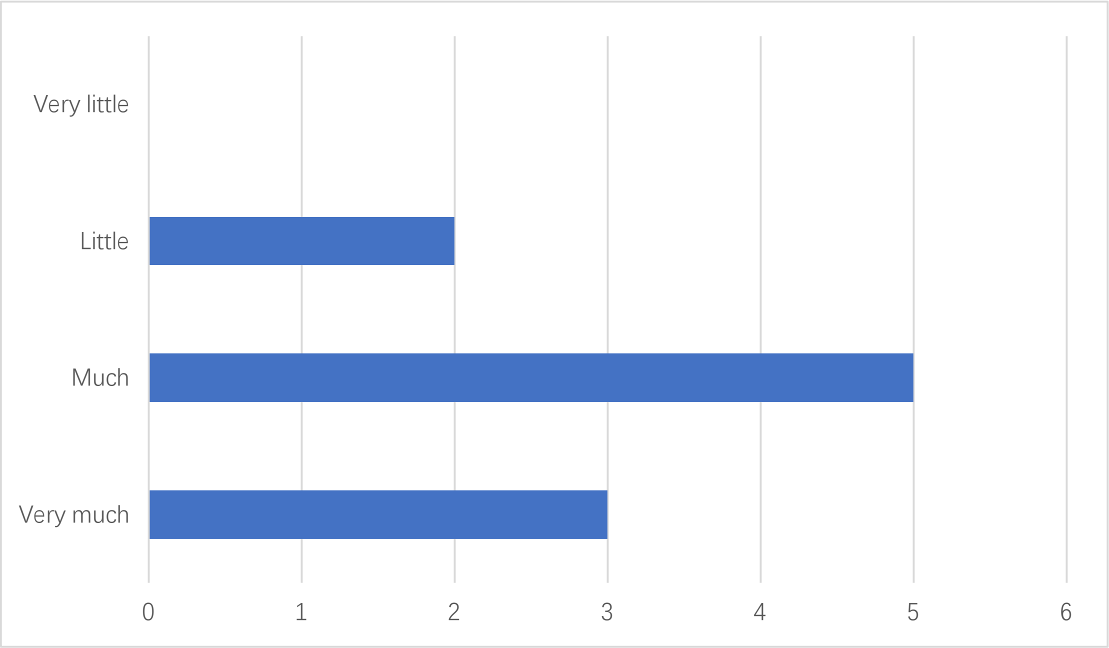

8. How long do you expect a rehabilitation training to last each time?

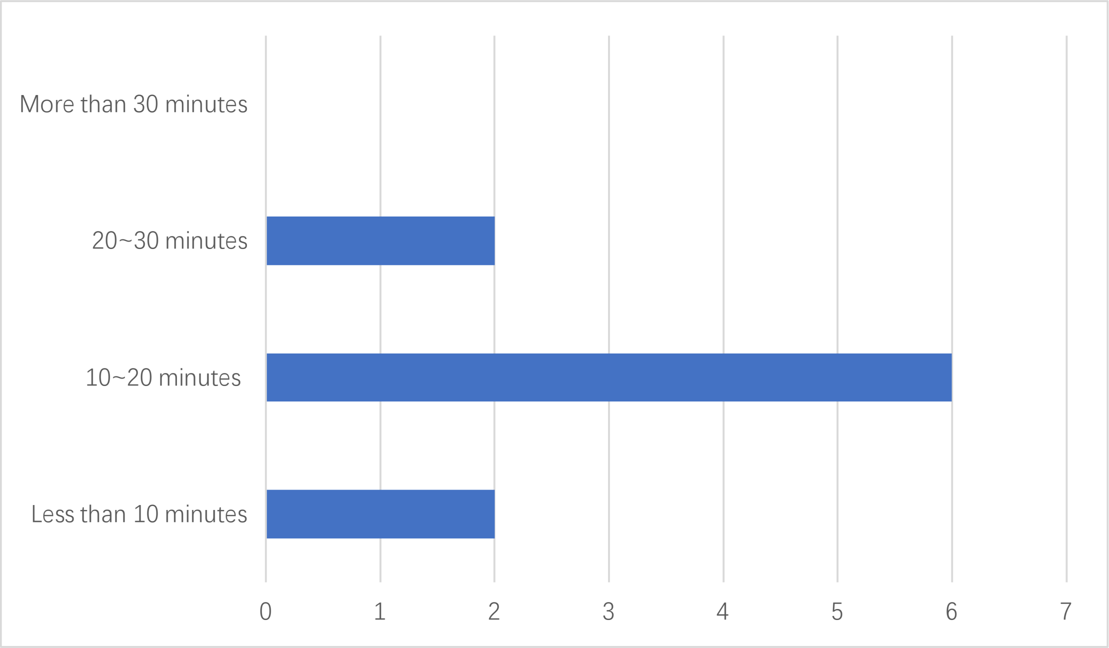
More than 30 minutes
20~30 minutes
10~20 minutes
Less than 10 minutes
0
1
2
3
4
5
6
7
