# Supplementary material for: The Effectiveness and Safety of Utilizing Mobile Phone–Based Programs for Rehabilitation After Lumbar Spinal Surgery: Multicenter, Prospective Randomized Controlled Trial
Source: JMIR Mhealth Uhealth. 2019 Feb 20;7(2):e10201. doi: 10.2196/10201 (PMC6404639; doi:10.2196/10201)
Supplement: Multimedia Appendix 3 [file mhealth_v7i2e10201_app3.docx]

Supplementary table 1: Baseline Characteristics of Lost to Follow Ups and those Met at the Last Follow Up in EH Group.

| Characteristic | Met at the last follow up EH group （n=60） | Lost to follow up EH group（n=24） | *P**  Value | Lost to follow up UC group（n=23） | *P #*  Value |
| --- | --- | --- | --- | --- | --- |
| Female, n (%) | 37(61.67) | 11(45.83) | 0.19 | 6(26.10) | 0.16 |
| Mean age (SD), y | 50.80(9.82) | 51.87(8.96) | 0.64 | 48.43(9.82) | 0.64 |
| Education status |  |  | 0.25 |  | 0.24 |
| High school or lower n (%) | 45(75.00) | 15(62.50) |  | 18(78.26) |  |
| college degree or higher n (%) | 15(25.00) | 9(37.50) |  | 5(21.74) |  |
| Currently employed | 50(83.30) | 18(75.00) | 0.38 | 21(91.30) | 0.14 |
| Marriage status |  |  | 0.36 |  | 0.76 |
| Married n (%) | 52(86.67) | 21(87.50) |  | 20(86.96) |  |
| Divorced n (%) | 3(5.00) | 3(12.5) |  | 2(8.69) |  |
| Single n (%) | 5(8.33) | 0(0.00) |  | 1(4.35) |  |
| Intervertebral discs involved in surgery |  |  | 0.46 |  | 0.15 |
| 1 disc n (%) | 27（45.00） | 9(37.50) |  | 15（65.22） |  |
| 2 discs n (%) | 27（45.00） | 14(58.33) |  | 7（30.43） |  |
| 3 discs n (%) | 6（10.00） | 1(4.17) |  | 1（4.35） |  |
| Mean ODI score (SD)^a^ | 55.18(15.21) | 51.54(15.11) | 0.32 | 57.78(13.37) | 0.32 |
| Mean VAS score (SD)^b^ | 58.58(14.95) | 55.54(14.90) | 0.40 | 63.00(13.60) | 0.40 |
| Mean Likert score (SD)^c^ | 63.35(14.31) | 60.08(14.05) | 0.34 | 62.57(12.42) | 0.34 |
| Mean EQ5D score(SD)^d^ | 33.73(12.54) | 37.71(12.57) | 0.19 | 34.04(16.03) | 0.19 |
| Mean SF36 GH score(SD) ^e^ | 13.30(6.08) | 14.33(5.94) | 0.61 | 12.39(4.94) | 0.61 |
| Mean SF36 PF score(SD) ^e^ | 21.22(8.19) | 22.29(10.06) | 0.48 | 20.09(4.94) | 0.48 |

*means comparison between lost to follow up and those met at the last follow up in EH group. #means comparison between EH group and UC group which lost to follow up.

Supplementary table 2: Baseline Characteristics between the Highest Compliance Group and Other Compliance Group.

| Characteristic | Highest compliance group（n=24） | Other compliance group（n=60） | P Value Between Groups |
| --- | --- | --- | --- |
| Female, n (%) | 16(66.70) | 32(53.3) | 0.26 |
| Mean age (SD), y | 34.13(14.62) | 35.17(11.82) | 0.09 |
| Education status |  |  |  |
| High school or lower n (%) | 20(83.33) | 40(66.67) | 0.13 |
| college degree or higher n (%) | 4(16.67) | 20(33.33) |  |
| Currently employed | 16(66.70) | 42(70.00) | 0.77 |
| Marriage status |  |  |  |
| Married n (%) | 22(91.70) | 51(85.00) |  |
| Divorced n (%) | 2(8.3) | 4(6.67) | 0.66 |
| Single n (%) | 0(0.00) | 5(8.33) |  |
| Intervertebral discs involved in surgery |  |  |  |
| 1 disc n (%) | 9(37.50) | 27（45.00） | 0.63 |
| 2 discs n (%) | 12(50.00) | 29（48.33） |  |
| 3 discs n (%) | 3(12.5) | 4（6.67） |  |
| Mean ODI score (SD)a | 55.75(18.00) | 53.50(14.02) | 0.54 |
| Mean VAS score (SD)b | 59.13(17.41) | 57.15(13.91) | 0.59 |
| Mean Likert score (SD)c | 63.46(17.04) | 62.00(13.08) | 0.67 |
| Mean EQ5D score(SD)d | 34.13(14.62) | 35.17(11.82) | 0.73 |
| Mean SF36 GH score(SD) e | 12.92(6.49) | 13.87(5.86) | 0.52 |
| Mean SF36 PF score(SD) e | 20.33(9.44) | 22.00(8.45) | 0.43 |

Supplementary table 3: Subgroup Analysis of Primary Outcomes Change from Baseline and 12 months Difference Outcomes

|  | UC group | | | EH group | | | |
| --- | --- | --- | --- | --- | --- | --- | --- |
| Characteristic | 12-24M Lost to follow up（n=9） | Met at the last follow up （n=61） | *P*  Value | 12-24M Lost to follow up（n=12） | Met at the last follow up （n=60） | *P*  Value | |
| Difference ODI score (SD) | -21.33(5.17) | -22.18(5.65) | 0.67 | -16.17(26.38) | -22.67(24.36) | | 0.41 |
| Difference VAS score (SD) | -23.00(4.15) | -21.79(6.03) | 0.56 | -15.50(26.75) | -21.57(25.86) | | 0.46 |

Supplementary table 4: Top Four Factors for Low Compliance Listed by Patients at 12 months

| Factors | n (%) |
| --- | --- |
| Lack of communication with doctors | 33（94.29） |
| Concern about the accuracy of the action | 20（57.14） |
| Symptom improvement was not obvious or even aggravated. | 27（77.14） |
| lack of motivation | 22（62.86） |
